# What do clinicians want? Interest in integrative health services at a North Carolina academic medical center

**DOI:** 10.1186/1472-6882-7-5

**Published:** 2007-02-09

**Authors:** Kathi J Kemper, Deborah Dirkse, Dee Eadie, Melissa Pennington

**Affiliations:** 1 Department of Pediatrics, Wake Forest University Baptist Medical Center, Winston-Salem, NC 27157, USA; 2 Department of Nursing, Wake Forest University Baptist Medical Center, Winston-Salem, NC 27157, USA; 3 Department of Strategic Planning, Wake Forest University Baptist Medical Center, Winston-Salem, NC 27157, USA

## Abstract

**Background:**

Use of complementary medicine is common, consumer driven and usually outpatient focused. We wished to determine interest among the medical staff at a North Carolina academic medical center in integrating diverse therapies and services into comprehensive care.

**Methods:**

We conducted a cross sectional on-line survey of physicians, nurse practitioners and physician assistants at a tertiary care medical center in 2006. The survey contained questions on referrals and recommendations in the past year and interest in therapies or services if they were to be provided at the medical center in the future.

**Results:**

Responses were received from 173 clinicians in 26 different departments, programs and centers. There was strong interest in offering several specific therapies: therapeutic exercise (77%), expert consultation about herbs and dietary supplements (69%), and massage (66%); there was even stronger interest in offering comprehensive treatment programs such as multidisciplinary pain management (84%), comprehensive nutritional assessment and advice (84%), obesity/healthy lifestyle promotion (80%), fit for life (exercise and lifestyle program, 76%), diabetes healthy lifestyle promotion (73%); and comprehensive psychological services for stress management, including hypnosis and biofeedback (73%).

**Conclusion:**

There is strong interest among medical staff at an academic health center in comprehensive, integrated services for pain, obesity, and diabetes and in specific services in fitness, nutrition and stress management. Future studies will need to assess the cost-effectiveness of such services, as well as their financial sustainability and impact on patient satisfaction, health and quality of life.

## Background

Use of complementary medicine is substantial and growing in the US [1-3]. As use has grown, terminology has evolved. The term "unconventional therapies", used in the early 1990's, referred to therapies not used in academic health centers, not taught in medical schools and not reimbursed by major insurers. This term evolved to "complementary and alternative" medical therapies (CAM) which was adopted by the National Institutes of Health (NIH) in 1998 when it created the National Center for Complementary and Alternative Medicine (NCCAM). NIH NCCAM defines CAM as a group of diverse medical and health care systems, practices, and products that are not presently considered to be part of conventional medicine." This definition has become problematic as therapies that were previously not part of conventional medicine are adopted into conventional settings such as teaching hospitals. For example, acupuncture has become routine in many pediatric pain treatment programs, as have biofeedback, hypnosis, guided imagery and music therapy [4]. Massage therapy is routinely used in neonatal settings [5]. Most major insurers reimburse for chiropractic services [6]. Therapies such as nutritional advice and advice about specific exercise may or may not be considered mainstream or complementary, depending on the content of the advice.

The formation of the Consortium of Academic Health Centers for Integrative Medicine (Consortium) in 2000 and the growing number of publications arising from the Cochrane Complementary Medicine Field (200 reviews as of 2006, accessed 8 January, 2007[7]) reflect the growing interest in integrating diverse therapies within the context of evidence-based comprehensive care at major academic medical centers. The Consortium defines integrative medicine as "the practice of medicine that reaffirms the importance of the relationship between practitioner and patient, focuses on the whole person, is informed by evidence, and makes use of all appropriate therapeutic approaches, healthcare professionals and disciplines to achieve optimal health and healing." Thus, the issue has been reframed, not CAM versus mainstream, but an integrated approach to comprehensive care that incorporates evidence-based therapies within the context of patient-centered care. Some might argue that this approach has simply renamed the adoption of evidence-based complementary therapies or good medicine as integrative medicine[8]. For this paper, we will refer to therapies as "CAM" in accordance with the tradition of authors of referenced papers and to specific therapies whenever possible.

Interest in specific therapies and integrative medicine varies regionally and in different demographic and clinical populations. CAM usage is reportedly lower in the southeast than other parts of the US, but even here the prevalence of using complementary therapies among users of conventional medicine reportedly exceeds 40% of adults [9]. In North Carolina in 2006, three academic health centers, University of North Carolina at Chapel Hill (UNC), Duke University School of Medicine, and Wake Forest University School of Medicine (WFUSM), were among the 36 members of the Consortium who use the term "integrative medicine" to refer to evidence-based integration of diverse therapies into comprehensive care.

Specifically, at WFUSM the strong interest in integrative medicine has been reflected in its research activities, e.g., nearly $16 million in funded research and 97 peer-reviewed publications on these topics in 2005–2006 (personal communication, WFUSM Program for Holistic and Integrative Medicine, 7/25/06). Integrative medicine is also incorporated into teaching within the medical school and physician assistant (PA) program at WFUSM which include extensive training in patient-centered care, development of a therapeutic relationship and case-based teaching that includes questions about dietary supplements, special diets, massage, meditation, yoga, acupuncture, chiropractic and homeopathy within existing teaching cases.

Many studies have assessed clinician attitudes and referral practices about CAM [10-40], but most have focused on outpatient services and on services distinct from those usually provided in an academic medical center. Fewer have focused on attitudes within academic medical centers [15,25,39,41-43]. Despite strong academic interest and rising consumer demand, little is known about the interest among our medical staff in offering clinical services marketed as comprehensive or integrative medicine.

The purpose of this study was to assess the interest of practicing clinicians at WFUSM in developing or augmenting integrative health services within the medical center. Here integrative services refers to integration of services which may have existed within the medical center for but utilized variably by different clinicians in different specialties (e.g., nutrition); newer services (e.g. massage therapy); services currently available only within research settings (e.g., yoga and mindfulness-based stress reduction); and services that might be considered in the future (e.g., biofeedback, expert consultation on herbs and dietary supplements, acupuncture and chiropractic).

## Methods

Cross-sectional surveys of staff physicians, nurse practitioners and physician assistants were conducted on-line between April 25 and July 6, 2006. An e-mail was sent by the Dean of the medical school asking clinical faculty to complete the anonymous survey on-line. The e-mail included a link to the on-line survey site. Four reminders were sent over one month to encourage completion. No incentives were offered for survey completion.

Physicians were eligible if they reported spending any time providing clinical care. Nurse practitioners and physician assistants were identified by the Human Resource Department of the medical school and hospital. Subjects were excluded if they spent 100% of their time in research and teaching (i.e., no clinical care), or if they were not employed by the medical school or hospital (e.g., community clinical faculty).

In addition to demographic questions, the survey included questions about two types of services: a) therapies themselves (e.g., dietary supplements such as herbs, fish oil or vitamins; therapeutic diets; therapeutic exercise; massage therapy; etc) and b) comprehensive services (e.g., multidisciplinary pain management; heart healthy lifestyle program; cancer patient support program). Some therapies and services currently exist within the institution (e.g., massage services and Cancer Patient Support Program), and some are potential future services and programs. Asking about existing services provided a benchmark against which to compare responses to potential services. The terms "CAM" and "alternative" medicine were avoided to minimize confusion and to facilitate comparison of diverse therapies within the familiar framework of comprehensive care. For each of the complementary therapies listed in the survey that may have been unfamiliar or confusing, we included links to the NIH NCCAM internet site defining those therapies (NIH NCCAM[44]), e.g., Reiki, yoga, Tai Chi, hypnosis, biofeedback, etc.

For individual services, we asked clinicians if they had provided it themselves or referred the patient to a provider within the medical center or the community, and how often would they anticipate referring a patient over the next 12 months. For example, under Therapies, Question 1 read: "Vitamins, minerals, other dietary supplements such as herbs, fish oil, glucosamine or melatonin:"

"In the past year, how often have you recommended or referred for this therapy?" "If you have referred/recommended a patient for this therapy at least once in the past year, where was the service provided (Wake Forest University Baptist Medical Center, WFUBMC, in the community or both)?" "In the next 12 months, how many times are you likely to use or refer patients to this therapy if provided at WFUBMC?" (Response categories ranged from 0 to 9 or more times.) For analysis purposes, we noted the number of respondents who anticipated making any referrals and the number making frequent (nine or more) referrals.

Similarly for the comprehensive service programs, some were already available at the Medical Center at the time of the survey. However, due to the size and complexity of the medical center, it is possible that physicians in some specialties are unaware of programs more pertinent to other specialties. We anticipated that fewer than 100% of respondents would refer patients to any of these programs given the diverse nature of respondents. For example, we thought it unlikely that pediatricians would refer patients to a "Comprehensive Back Pain" program. Questions about comprehensive services followed questions about specific therapies to help respondents understand that comprehensive services include diverse therapies in addition to medications and surgery. For example, "In the next 12 months, how many times are you likely to use or refer patients to each of the following: a. Multidisciplinary pain management."

Answers for questions were scored as the percentage of respondents who reported any referrals (greater than 0) and the percentage who would refer 9 or more times. Open text fields were also available for each question for respondents to write comments.

The survey was written by a programmer who was blinded to study questions using the Empliant survey writing software. The Empliant software was able to anonymously tabulate the results. Altogether the instrument contained 62 questions and could be completed on-line in less than 20 minutes.

Analysis included descriptive statistics only. There were no a priori hypotheses. The study was conducted to generate information for service development and strategic planning rather than hypothesis testing.

## Results

Of 537 full-time and part-time physicians with admitting privileges, responses were received from 125 (24%). Response rates were higher among nurse practitioners (NP 23/47, 48%) and physician assistants (PAs 21/45, 46%). Of the 169 respondents, only two were less than 30 years old, and only 12 respondents were over 60 years old (Table 1). Respondents represented the spectrum of clinical departments and programs at the medical center including pediatrics, internal medicine, surgery, psychiatry, obstetrics/gynecology, geriatrics and numerous specialties within these fields.

**Table 1:** Respondent Characteristics

**Characteristic**	**Physicians**	**Nurse Practitioners**	**Physician Assistants**	**TOTAL**
Number respondents/eligible	125/537 (24%)	23/47 (48%)	21/45 (46%)	169/629 (26%)
				
*Age*				
< 30 years old	1	0	1	2
30–39	33	4	2	39
40–49	47	10	5	62
50–59	33	9	9	51
60 or greater	10	0	2	12
				
*Departments*				
Anesthesiology	6			6
Critical care	3	1		4
Dermatology	6		1	7
Emergency medicine	6	1		7
Family Medicine	5	1	1	7
Hematology/Oncology and Radiation Oncology	12	2	5	19
Internal Medicine including Cardiology	26	5	3	34
Neurology	5	1		6
Ob-Gyn	5			5
Pediatrics	13	7	1	21
Psychiatry	5			5
Surgery, any type (general, CT, neuro, vascular, ophtho, ortho, uro, plastic etc)	12	4	6	22
Other	8		1	9

Among the individual services listed on the survey, clinicians were most interested in having a therapeutic exercise program, followed by consultation services for vitamins/minerals and other dietary supplements; therapeutic massage; environmental therapies; prayer, spiritual healing, ceremony or ritual (pastoral care); and acupuncture; for each of these services, more than 50% of respondents would anticipate making at least one referral in the next 12 months. (Table 2). For example, for therapeutic exercise, 77% of clinicians had made a referral in the past year, nearly half of these to programs in the community (such as the YMCA). If such services were to be offered at the medical center, 80% of clinicians would anticipate referring in the next 12 months, with 58% referring 9 or more times. Less than 40% of clinicians anticipated referring patients for chiropractic, Reiki, or homeopathy, even if they were offered at the medical center.

**Table 2:** Therapeutic recommendations or referrals for specific ancillary services

**Therapy**	**MD**	**NP**	**PA**	**TOTAL**
N respondents	125	23	21	169
				
***Therapeutic exercise, e.g. swimming, walking, yoga, Tai Chi***	96/124 (77%)	16/23 (69%)	18/21 (80%)	130/168 (77%)
Refer to community only	39/90 (43%)	6/16 (38%)	8/17 (47%)	53/123 (43%)
Would refer if offered here	95/120 (79%)	16/20 (80%)	18/21 (80%)	129/161 **(80%)**
Would refer 9+/yr	70/120 (58%)	11/20 (55%)	13/21 (62%)	94/161 (58%)
				
***Vitamins, minerals, and other dietary supplements such as herbs, fish oil, glucosamine or melatonin***	82/125 (66%)	15/23 (65%)	16/21 (76%)	113/169 (67%)
Refer to community only	22/73 (30%)	6/15 (40%)	7/15 (47%)	35/103 (33%)
Would refer if offered here	82/118 (69%)	14/20 (70%)	15/21 (71%)	111/159 **(69%)**
Would refer 9+/yr	39/118 (33%)	7/20 (35%)	8/21 (38%)	54/159 (33%)
				
***Therapeutic Massage or Bodywork***	60/124 (48%)	11/23 (48%)	14/21 (67%)	85/168 (50%)
Refer to community only	39/59 (66%)	4/11 (36%)	5/15 (33%)	48/85 (56%)
Would refer if offered here	72/112 (64%)	14/21 (67%)	15/19 (79%)	101/152 **(66%)**
Would refer 9+/yr	22/112 (20%)	2/21(10%)	5/19 (26%)	29/152(19%)
				
***Environmental Therapy, e.g. bright lights, phototherapy, music, heat, ice, magnets, etc***.	44/123 (34%)	11/23 (48%)	11/21 (52%)	66/167 (39%)
Refer to community only	10/44 (23%)	4/11 (36%)	4/11 (36%)	18/66 (27%)
Would refer if offered here	58/111 (52%)	12/21 (57%)	13/18 (72%)	83/150 **(55%)**
Would refer 9+/yr	21/111 (19%)	1/21 (5%)	6/18 (33%)	28/150 (18%)
				
***Prayer/spiritual counseling, ceremony or ritual***	53/124 (43%)	13/23 (56%)	10/21 (48%)	76/168 (45%)
Refer to community only	28/54 (52%)	4/13 (31%)	5/10 (50%)	37/77 (48%)
Would refer if offered here	55/114 (48%)	15/19 (79%)	10/18 (56%)	80/151 **(52%)**
Would refer 9+/yr	24/114(21%)	6/19(31%)	5/18(28%)	5/151(23%)
				
***Acupuncture, acupressure or related therapies***	39/123 (32%)	2/23 (10%)	7/20 (35%)	48/166 (28%)
Refer to community only	36/41 (88%)	2/23 (10%)	7/7 (100%)	45/71 (63%)
Would refer if offered here	58/111 (52%)	8/19 (42%)	9/17 (53%)	75/147 **(51%)**
Would refer 9+/yr	4/111 (4%)	0%	4/17 (24%)	8/128 (6%)
				
***Chiropractic or osteopathic adjustments***	31/123 (25%)	5/22 (23%)	9/21 (43%)	45/166 (27%)
Refer to community only	26/34 (76%)	4/4 (100%)	8/9 (89%)	38/47 (80%)
Would refer if offered here	38/109 (35%)	7/18 (39%)	8/17 (47%)	53/144 **(36%)**
Would refer 9+/yr	4/109 (4%)	1/18(6%)	3/17 (18%)	8/144(5%)
				
***Reiki, healing touch, therapeutic touch, QiGong***	15/120 (12%)	4/23 (17%)	3/21 (14%)	22/164 (13%)
Refer to community only	11/16 (69%)	1/4 (25%)	3/3 (100%)	15/23 (65%)
Would refer if offered here	31/106 (29%)	9/18 (50%) 0	7/17 (41%)	47/141 **(33%)**
Would refer 9+/yr	1/106 (1%)	%	3/17 (18%)	4/123 (3%)
				
***Homeopathy***	3/122 (2%)	2/23 (9%)	3/21 (14%)	8/166 (0.5%)
Refer to community only	4/7 (57%)	1/2 (50%)	3/3 (100%)	8/12 (66%)
Would refer if offered here	18/110 (16%)	7/18 (39%)	4/15 (26%)	29/143 **(20%)**
Would refer 9+/yr	0%	0%	1/15 (7%)	1/15 (7%)

NOTE: not all respondents answered every question. Percentages refer to those who gave the described answer among those who answered this question

When asked comprehensive service programs they would likely refer to in the next 12 months if offered at the medical center (Table 3), an overwhelming number would refer at least once a year to a multidisciplinary pain management program (84%), a program offering comprehensive nutritional assessment and advice (84%), a program offering healthy lifestyle promotion for obesity (80%) a fit for life program (exercise and fitness, 76%), a healthy lifestyle promotion program for diabetes (73%), a comprehensive stress management program (73%), and a healthy lifestyle promotion program for cardiovascular health (71%). There was also substantial support for a pharmacy program offering clinicians support for clinical questions about herbs and dietary supplements (68%), a comprehensive back pain program (66%), an integrative headache management program (63%) and a cancer patient support program (62%).

**Table 3:** Anticipated referrals to comprehensive services offered at WFUBMC in next 12 months

**Service**	**MD**	**NP**	**PA**	**TOTAL**
N	N = 125	N = 23	N = 21	169
				
***Multidisciplinary Pain Management***				
Any	106/123 (86%)	16/23 (70%)	19/21 (90%)	141/167(**84%)**
9 or more times	52/123 (42%)	7/23 (30%)	10/21 (48%)	69/167 (41%)
				
***Comprehensive Nutritional Assessment and Advice***				
Any	99/116 (85%)	18/22 (88%)	16/20 (80%)	133/158 (**84%)**
9 or more times	43 (37%)	9/22 (41%)	8/20 (40%)	60/158(37%)
				
***Obesity Healthy Lifestyle Promotion***				
Any	99/119 (83%)	15/23 (65%)	18/21 (86%)	132/163 (**80%)**
9 or more times	53/119 (45%)	7/23 (30%)	8/21 (38%)	68/163 (41%)
				
***Fit for Life (Exercise/Fitness Program)***				
Any	90/116(77%)	15/23 (65%)	17/21 (81%)	122/160(**76%)**
9 or more times	43 (37%)	6/23 (26%)	10/21 (48%)	59/160(36%)
				
***Diabetes Healthy Lifestyle Promotion***				
Any	86/118 (73%)	16/23 (70%)	17/21 (81%)	119/162 (**73%)**
9 or more times	38/118 (32%)	6/23 (26%)	7/21 (33%)	51/162 (31%)
				
***Psychology support for biofeedback, hypnosis, Mindfulness-based Stress Reduction, other stress management***				
Any	92/119 (77%)	13/22 (60%)	14/21 (67%)	119/162 (**73%)**
9 or more times	31 (26%)	2/22 (9%)	7/21 (33%)	40/162 (24%)
				
***Heart Healthy Lifestyle Promotion***				
Any	81/116 (70%)	16/23(70%)	18/21 (86%)	115/160 **(71%)**
9 or more times	39/116 (34%)	7/23(30%)	6/21 (29%)	52/160 (32%)
				
***Pharmacy Support for questions about herbs and supplements***				
Any	80/119 (67%)	16/23 (70%)	16/21 (76%)	112/163 (**68%)**
9 or more times	23 (19%)	6/23 (26%)	8/21 (38%)	37/163 (22%)
				
***Comprehensive Back Pain***				
Any	84/123 (68)	12/23 (52%)	15/21 (71%)	111/167 (**66%)**
9 or more times	32/123 (26%)	2/23 (9%)	9/21 (43%)	43/167 (25%)
				
***Integrative Headache management***				
Any	80/121 (66%)	12/23 (52%)	13/21 (62%)	105/165 (**63%**)
9 or more times	23/121 (19%)	1/23(4%)	6/21 (29%)	30/165(18%)
				
***Cancer Patient Support Program***				
Any	76/117 (65%)	10/23 (44%)	15/21 (71%)	101/161(**62%**)
9 or more times	25/117 (21%)	3/23 (13%)	5/21 (24%)	33/161(20%)
				
***Stroke Recovery Program***				
Any	60/120 (50%)	12/23 (52%)	12/20 (60%)	84/163(**51%)**
9 or more times	10/120 (8%)	2/23 (9%)	2/20 (10%)	14/163(8%)

There were numerous comments in the free text fields. Many respondents were very enthusiastic in their responses, often based on their personal experiences:

"Music therapy is fantastic. I spent some time at X in a post op ward, and they had musicians come to the floors and play, and I can guarantee that not only did the visitors appreciate it, so did the patients, which was the important reason."

"I have degenerative arthritis and started taking yoga myself when Vioxx went off the market. I am so thoroughly convinced of its benefits that I think it ought to be offered as a prescribed form of physical and mental therapy. .... It has changed my life."

"We NEED to have a multidisciplinary pain management and back pain clinic. .. My patients would receive much better care if such a clinic were available."

"We need more psychologists here to provide these therapies. Too time consuming for docs to do it."

"Meditation is very important in a person's life, and to teach or lead someone in it is a very valuable asset."

"I really wish massage was readily available for inpatients." "We need it here."

Several clinicians recommended specific acupuncturists, yoga teachers, exercise programs, Tai Chi instructors, and massage therapists in the community with whom they'd had good relationships. Others listed specific dietary supplements they already recommend for their patients (e.g., multivitamins, vitamins C, D, E, folic acid, calcium, zinc, fish oil, glucosamine, melatonin and probiotics).

A few respondents were cautious and expressed concerns about the scientific evidence for effectiveness, the need for additional education about the therapy or the need for the therapy to be provided only under certain circumstances, even for some therapies currently offered at the institution. For example, regarding acupuncture "Need scientific info regarding efficacy," and regarding special diets, "More CME on this topic, please."

Some respondents felt that at least some of the listed therapies are really mainstream because they:

"have ICD9 and CPT codes.... And are covered expenses by insurers."

"The ketogenic and Atkins diets are both used in the treatment of epilepsy."

"We prescribe special diets for patients with swallowing dysfunction."

"Low fat, high fiber diets are a routine part of clinical practice for many dealing with vascular disease."

"I often recommend low glycemic index diets, Mediterranean style or Ornish type intake."

"Talking about diet is pretty mainstream medicine."

Similarly, "Vitamins and minerals are pretty common recommendation." An ophthalmologist stated, "The only proven clinical benefit from vitamin treatment is for macular degeneration." While an endocrinologist averred, "I advise the use of vitamin D, omega three fish oils, calcium and multivitamin every time I am in clinic." Another specialist wrote, "Vitamins such as folate are a routine part of clinical practice for many dealing with vascular disease."

Furthermore, environmental therapies are "routine in the NICU environment to the extent possible, e.g., low lights, light cycling, and noise control." They (phototherapy) were also "routinely used" in dermatology, while "heat and icing are a routine part of clinical practice for clinicians dealing with musculoskeletal diseases." Similarly, "I treat pain and other distressful symptoms, and I fully advocate for non-pharmacologic modalities of therapy." And

Stress management therapies, "I do recommend relaxation, breathing exercises and prayer (meditation) as a way of stress management." "I discuss general stress management in my clinics."

However, respondents from other specialties sometimes stated directly opposing negative or skeptical views, (e.g., regarding environmental therapies), "This is not appropriate medical therapy."

Few respondents had negative or disparaging comments about therapies listed on the survey. For example, in regard to vitamins and minerals, one physician wrote "We are scientists first, artists second." The most consistently negative comments among a larger group of respondents were expressed about chiropractic and homeopathy,

"This (chiropractic) should NEVER be recommended by an allopathic MD. Chiropractic is pseudoscience."

"I don't believe that homeopathy works. I would never recommend it."

Access to and payments for care were cited as concerns by several respondents:

"Would I use these programs, you bet, but working at the __ Clinic, 40% of my patients are self pay. The rest are Medicare and Medicaid. We have only about 5% that are private insurance. So my utilization would to a large extent be based on cost."

"It depends on the cost to the patients as to how much my patients can utilize these therapies."

And most succinctly, "Who pays?"

Several respondents wrote that although a specific therapy or comprehensive service system was not relevant to their particular specialty, they would nonetheless like to see it offered at the medical center. For example, regarding acupuncture,

"It is not applicable to me in my clinical setting (neonatology), but I hope this is a modality that the medical center will take a greater part in, especially for cancer and chronic pain patients."

## Discussion

This survey of clinicians at a North Carolina academic medical center supports findings from previous surveys of clinicians and informs the planning of future services. Most physicians, such as those in our study and others, are interested in and refer to diverse services and would like to see integration of a variety of evidence-based services at their institution [25,30,41]; the greatest interest is in therapies with which they have had personal experience or professional training such as lifestyle therapies (nutrition and exercise) and mind-body therapies [11,13,19,45,46].

In this study, the highest level of support was for an integrated, comprehensive pain treatment program available for referrals from across the institution. This is similar to other surveys which have ranked pain as the most common reason for physician referral for CAM services [15,26,47]. Pain is also a common reason that patients seek CAM services [4,48-50]. This survey also showed a very strong interest in comprehensive programs for obesity and diabetes, which have not been noted in previous surveys. It is possible that future studies may reveal this interest elsewhere as the obesity epidemic continues to grow. It is also possible that numbers were higher in this survey than previous surveys because questions were asked about specific therapies and comprehensive programs for specific conditions rather than labeling them as CAM.

Most physicians view at least some therapies previously referred to as CAM as mainstream rather than alternative [22,30,51]. Our results are similar to others in that lifestyle therapies such as advice about exercise, fitness, nutrition, and stress management/relaxation are now considered mainstream [11,12,31,41]. In our sample, nutrition services were viewed by respondents as mainstream whereas in Crock's 1999 survey, food and special diets were rarely used or recommended by physicians [18]. In other recent surveys, more than half of physicians referred patients for massage, chiropractic and acupuncture [13,14]. Similarly, in our sample, 50% or more clinicians had referred patients to massage or stress management programs, but only slightly more than 25% had referred patients for acupuncture or chiropractic. These data support suggestions that as the field rapidly evolves, changes in terminology are needed to more accurately reflect specific services, rather than lumping groups of therapies under heterogeneous terms such as CAM.

Unlike most other surveys about physician attitudes, this one included a question about the desirability of having expert consultation about herbs and other dietary supplements. Two thirds of our respondents reported having recommended dietary supplements in the past year. A consult service providing expert information about dietary supplements was viewed as desirable by the majority of respondents, perhaps reflecting clinicians' growing awareness of their patients' use of dietary supplements and the potential for side effects and supplement-medication interactions [52-56].

Integrative care is supplanting the dichotomy between mainstream and CAM for some clinicians as well as patients [21,57-62]. Most clinicians have been asked about CAM therapies by patients and are aware that patients are using these therapies [24,34,63]. Physicians are most comfortable in referring patients for therapies they have used personally [16,23,36] and those for which they've received professional training and are most knowledgeable [11,42,59,64]. For example, therapies that are not commonly taught and which appear to be inconsistent with current scientific understanding, such as homeopathy and biofield therapies (e.g., magnets and therapeutic touch) appear to have the least support among medical staff [45,51].

Researchers and policy makers should be aware that not all clinicians view therapeutic options similarly. For example in our survey, pediatricians (including neonatologists) reported providing environmental therapies (cycling light, reducing noise, using phototherapy) and recommending massage therapy whereas other specialists did not often recommend environmental changes or they restricted them to one modality, e.g., light therapy for certain dermatologic conditions. Other specialists were frankly skeptical about therapies viewed as routine by others. For example, most specialists routinely recommended dietary supplements, whereas others did not and questioned the need for ever using them.

The different attitudes and practices of different specialists in this survey are consistent with previous surveys, and suggest important gaps in communication and the need for additional education. Many physicians in our survey appeared to be unaware of the availability of nutrition services and massage services throughout the institution or the Cancer Patient Support Program available through the Comprehensive Cancer Center or fitness advice and programs available through physical therapy. We suspect these kinds of gaps are not unique to our institution, and that they demonstrate the need for better intra-institutional communication.

As interest grows in integrating diverse therapies into mainstream practice, there is a tremendous need for professional education about them. Our results are consistent with earlier studies reporting strong interest among physicians in receiving additional training about therapies previously known as CAM [19,20,24], e.g., more than 70% of practicing physicians want additional training in CAM [11,24,29,34,40]. Although most medical schools and a growing number of residency programs now provide some training in CAM [25,62,65,66], physicians who trained more than ten years ago may not have received formal training about these topics during medical school or residency training. This presents a large market for institutions offering Continuing Medical Education.

In addition, medical centers that are planning new or augmented clinical services need to consider access to care and payment strategies to ensure that these services are financially sustainable and equitably available to those who need them. Several respondents in this survey indicated their interest in providing integrated services, but concern about their patients' ability to pay for them. Reimbursement by insurance is changing, but in North Carolina, it does not provide universal coverage for services such as nutrition and fitness counseling, stress management, therapeutic massage or acupuncture, particularly when these services are provided by non-physician clinicians. Most insurance does cover chiropractic services, but few physicians appear eager to provide these services within an academic medical center.

Most research on CAM has assessed patient use of therapies, and there appears to be some incongruence between what patients want, what insurers cover, what physicians recommend, and what hospitals provide. Among patients, the most commonly used so-called CAM therapies include prayer, dietary supplements such as herbs and vitamins, mind-body therapies (such as relaxation techniques, meditation and breathing techniques), chiropractic, and massage [1]. Insurers, on the other hand, cover chiropractic, acupuncture, and biofeedback more often than home remedies, dietary supplements, or prayer [67]. More than 25% of hospitals offer CAM therapies, most often to outpatients. Among the hospitals that offer complementary services, the services most often provided on an outpatient basis include massage (71%); tai chi, yoga or chi gong (48%); relaxation training (43%); acupuncture (39%); guided imagery (32%); and therapeutic touch (30%). Among the hospitals providing complementary services to inpatients, the most common services are: massage (37%); music/art therapy (26%); therapeutic touch (25%); guided imagery (22%); relaxation training (20%) and acupuncture (12%) [68].

Typical of physician surveys, this study had a low response rate, which may lead to over-representation of responses among those with the strongest feelings about an issue. Furthermore, the survey included only one medical center at one point in time, further limiting generalizability. However, it is reassuring that the major findings of this survey are consistent with similar surveys at other medical centers over the past five years. Although the study included a broad range of specialties reflective of the institution, we did not analyze data by specialty, age or gender of respondents because the purpose was to aid strategic planning rather than test hypotheses and because the number of physicians responding in each specialty was too small for meaningful comparisons. Future research may target particular groups of clinicians as services are developed for individual departments and programs. Finally, the types of therapies and service lines listed in this survey may not have been exhaustive, and researchers in areas in which other services are used may need to ask additional questions. Similarly, some questions may have covered overlapping topics, and future planners may wish to distinguish therapies more precisely before implementing new service lines.

## Conclusion

This study demonstrates clinicians' interest in more comprehensive services particularly for the treatment of pain (both inpatient and outpatient) and obesity and the development of new services such as expert consultation on dietary supplements and stress management. There is also an urgent need for continuing education (CME) about complementary therapies; this represents a program development opportunity. In order for these services to be sustainable and accessible, work is needed to ensure that the major insurance programs expand their coverage of services, particularly those that are provided by non-physician clinicians. Finally, as services are developed and implemented additional research will be needed to evaluate the cost-effectiveness of integrative services and their impact on patient satisfaction, health and quality of life.

## Competing interests

The author(s) declare that they have no competing interests.

## Authors' contributions

KK conceived of the study, designed survey questions and drafted the manuscript.

DD reviewed survey questions, conducted data analysis and edited the manuscript.

DE reviewed survey questions and edited the manuscript.

MP conducted the survey, compiled the data and reviewed the manuscript.

All authors read and approved the final manuscript.

## Pre-publication history

The pre-publication history for this paper can be accessed here:


